# Supplementary material for: Prevalence and Social Determinants of Food Insecurity among College Students during the COVID-19 Pandemic
Source: Nutrients. 2020 Aug 20;12(9):2515. doi: 10.3390/nu12092515 (PMC7551032; doi:10.3390/nu12092515)
Supplement: Supplementary file 1 [file nutrients-12-02515-s001.zip › nutrients-898186-supplementary.docx]

**Table S1.** Frequency of responses to individual questions on the 2-item Food Sufficiency Screener (*n* = 502).

| **Variables** | **Survey Unweighted** | | **Survey Weighted** |
| --- | --- | --- | --- |
|  | ***n*** | **%** | **%** |
| Q1. Did you ever run short of money and try to make your food or your food money go further? |  |  |  |
| Yes | 186 | 37.1 | 38.9 |
| No | 313 | 62.4 | 60.8 |
| Decline to answer | 3 | 0.6 | 0.3 |
| Q2. Which of these statements best describes the food eaten in your household? |  |  |  |
| Enough of the kinds of food we want to eat | 216 | 43.0 | 42.5 |
| Enough but not always the kinds of food we want to eat | 248 | 49.4 | 48.6 |
| Sometimes not enough to eat | 34 | 6.8 | 8.2 |
| Often not enough to eat | 4 | 0.8 | 0.7 |

Note: All questions were prefaced with “In the last 30 days.” Weighted values are weighted for sex and race/ethnicity.

**Table S2**. Frequency of responses to questions on the 6-Item USDA Food Security Survey Module (*n* = 319).

| **Variables** | **Survey Unweighted** | | **Survey Weighted** | |
| --- | --- | --- | --- | --- |
|  | ***n*** | **%** | **%** |  |
| Q1. Did you ever reduce the size of your meals or skip meals because there was not enough money for food? |  |  |  |  |
| Yes | 119 | 37.3 | 38.2 |  |
| No | 188 | 58.9 | 58.3 |  |
| Decline to answer | 12 | 3.8 | 3.5 |  |
| Q2. How often did you reduce the size of your meals or skip meals because there was not enough money for food? |  |  |  |  |
| < 3 days during the last 30 days | 223 | 72.9 | 73.3 |  |
| ≥ 3 days during the last 30 days | 83 | 27.1 | 26.7 |  |
| Q3. Did you ever eat less than you felt should because there was not enough money for food? |  |  |  |  |
| Yes | 141 | 44.2 | 46.7 |  |
| No | 172 | 53.9 | 51.1 |  |
| Decline to answer | 5 | 1.6 | 2.0 |  |
| Do not know | 1 | 0.3 | 0.2 |  |
| Q4. Did you experience hunger and did not eat because there was not enough money for food? |  |  |  |  |
| Yes | 60 | 18.8 | 20.4 |  |
| No | 248 | 77.7 | 75.8 |  |
| Decline to answer | 7 | 2.2 | 2.7 |  |
| Do not know | 4 | 1.3 | 1.1 |  |
| Q5. The food that I/we bought just did not last, and I/we did not have enough money to get more. |  |  |  |  |
| Often true | 12 | 3.8 | 3.6 |  |
| Sometimes true | 124 | 38.9 | 40.3 |  |
| Never true | 173 | 54.2 | 53.0 |  |
| Do not know | 9 | 2.8 | 2.7 |  |
| Decline to answer | 1 | 0.3 | 0.4 |  |
| Q6. I/we could not afford to eat balanced meals. |  |  |  |  |
| Often true | 53 | 16.6 | 17.1 |  |
| Sometimes true | 123 | 38.6 | 38.0 |  |
| Never true | 135 | 42.3 | 42.3 |  |
| Do not know | 7 | 2.2 | 2.4 |  |
| Decline to answer | 1 | 0.3 | 0.2 |  |

Note: All questions were prefaced with “In the last 30 days” and included “you/other adults in your household” when asking the survey questions. Weighted values are weighted for sex and race/ethnicity.

**Table S3.** Predictors of food insecurity during the COVID-19 pandemic using unweighted data.

| **Effect** | **Odds Ratio Estimate**  **(95% CI)** | ***p*-value** |
| --- | --- | --- |
| Race/ethnicity |  |  |
| Black vs. White | 1.79 (0.90, 3.57) | 0.09 |
| Hispanic vs. White | 2.07 (1.19, 3.60) | 0.01 |
| Asian/Pacific Islander vs. White | 2.12 (1.14, 4.97) | 0.02 |
| Other race/ethnicity vs. White | 1.20 (0.51, 2.82) | 0.67 |
| BMI (kg/m^2^) |  |  |
| Overweight/obese vs normal weight | 1.29 (0.84, 1.97) | 0.25 |
| Current living arrangement |  |  |
| Live with roommates and/or spouse vs. I live alone | 0.86 (0.40, 1.84) | 0.69 |
| Live with parents or other relatives vs. I live alone | 0.58 (0.26, 1.28) | 0.18 |
| Live with my children only vs. I live alone | 1.81 (0.59, 5.58) | 0.29 |
| COVID-19 pandemic impacted current living arrangement? Yes vs. no | 2.86 (1.78, 4.61) | < 0.001 |
| Change in current employment status due to COVID-19 pandemic |  |  |
| Furloughed vs. income/employment unchanged | 3.58 (1.87, 6.86) | < 0.001 |
| Laid off vs. income/employment unchanged | 3.49 (1.62, 7.52) | 0.001 |
| Lost part-time shift work vs. income/employment unchanged | 4.65 (2.43, 8.89) | < 0.001 |
| Other changes in income^a^ vs. income/employment unchanged | 3.60 (2.05, 6.31) | < 0.001 |

Notes: ^a^ Other included: (1) reduced work hours, (2) resigned or quit job due to unsafe working conditions and/or presence of a pre-existing condition, and (3) spouse or parent lost their job or were furloughed. BMI: Body Mass Index.
